# Nicotinic and Folic Acid Supplementation Improves Cryopreserved Ram Sperm Quality, Potentially Through Modulation of *OGG1* and *ROMO1* Expression and DNA Methylation

**DOI:** 10.3390/ijms27188422

**Published:** 2026-09-21

**Authors:** Mehmet Melih Yılmaz, Tugaycan Cimen, Sena Ardicli, Oguzhan Huraydin, Elif Gokce, Deniz Karakci, Ahmet Aktar, Zekariya Nur, Muhammed Duman, Burcu Ustuner

**Affiliations:** 1 Department of Reproduction and Artificial Insemination, Faculty of Veterinary Medicine, Bursa Uludag University, 16059 Bursa, Türkiye; mehmetmelihyilmaz96@gmail.com (M.M.Y.); tugaycancimen@gmail.com (T.C.); ohuraydin@ku.edu.tr (O.H.); ahmet.aktar@balikesir.edu.tr (A.A.); nurzek@uludag.edu.tr (Z.N.); 2 Swiss Institute of Allergy and Asthma Research (SIAF), University of Zurich, CH-7265 Davos, Switzerland; sardicli@uludag.edu.tr; 3 Department of Genetics, Faculty of Veterinary Medicine, Bursa Uludag University, 16059 Bursa, Türkiye; 4 Department of Reproduction and Artificial Insemination, Faculty of Veterinary Medicine, Namık Kemal University, 59030 Tekirdag, Türkiye; a.elif.gokce@gmail.com; 5 Department of Biochemistry, Faculty of Veterinary Medicine, Namık Kemal University, 59030 Tekirdag, Türkiye; dkarakci@nku.edu.tr; 6 Department of Aquatic Animal Diseases, Faculty of Veterinary Medicine, Bursa Uludag University, 16059 Bursa, Türkiye; mduman@uludag.edu.tr

**Keywords:** cryopreservation, mitochondrial membrane potential, DNA integrity, antioxidants

## Abstract

This study aimed to evaluate the effects of nicotinic acid (NA) and folic acid (FA) as antioxidants on post-thaw spermatological characteristics and *OGG1*/*ROMO1* gene expression and methylation. Collected ram semen was pooled and divided into four equal aliquots, diluted with different dose groups: Control, 10 mM NA with no FA (NA10), 10 mM NA + 50 nM FA (NA10FA50) and 20 mM NA + 50 nM FA (NA20FA50). Although not statistically significant (*p* > 0.05), the NA10FA50 (53.34%) group showed the highest numerical mean motility, whereas the control group (43.44%) showed the lowest numerical mean motility. There were no statistical differences in terms of plasma membrane and DNA integrity, malondialdehyde and total antioxidant capacity rates. The lowest acrosomal damage was obtained in the NA10FA50 group (36.50%), which was only statistically different from the control (48.00%) (*p* < 0.05). The lowest mitochondrial membrane potential was observed in the control group (13.80%) (*p* < 0.05). NA10FA50 and NA20FA50 altered OGG1 gene expression, which was accompanied by corresponding changes in DNA methylation, suggesting a potential epigenetic regulatory relationship. *ROMO1* expression was significantly higher in the NA10 compared to the NA10FA50 (*p* < 0.01), accompanied by differences in DNA methylation patterns (*p* < 0.05). This study suggests that the addition of 10 mM NA or a combination with 50 nM FA showed beneficial effects on the post-thaw quality of ram semen, particularly with respect to acrosomal integrity and mitochondrial membrane potential and selected molecular markers.

## 1. Introduction

Cryopreservation induces molecular disruptions in spermatozoa that negatively affect their structure, biochemistry, and function, leading to reduced motility, viability, and fertilization efficiency; this effect is especially significant in ram spermatozoa owing to the characteristically low-cholesterol/phospholipid content of their plasma membranes [1]. Despite the development of various extenders and freezing protocols for ram semen, the fertility outcomes achieved with frozen-thawed samples remain lower than those obtained using fresh semen or through natural mating [2,3,4]. Cryopreservation-induced cold shock disrupts the structural integrity of spermatozoa membranes, thereby increasing their susceptibility to oxidative stress (OS). Reactive oxygen species (ROS), as primary mediators of OS, impair membrane fluidity and trigger lipid peroxidation cascades, ultimately compromising cellular function. These oxidative alterations impair the bioenergetic functions of the spermatozoon tail by reducing mitochondrial membrane potential (MMP) [2,5]. In addition, OS is associated with increased DNA fragmentation and the induction of apoptosis [5,6]. Studies have indicated that adding antioxidant compounds to ram semen freezing extenders can improve post-thaw sperm quality by combating the detrimental effects of ROS [7,8,9,10].

Nicotinic acid (NA) is a vitamin B3 derivative and forms the functional part of Nicotinamide Adenine Dinucleotide (NAD), which is one of the coenzymes in oxidation and reduction reactions in cellular metabolism [11,12]. Due to the fact that NA is involved in the functional part of enzymes in cellular oxidative defense reactions, it has been reported that lipid peroxidation is formed in its deficiency [13]. Besides its metabolic roles, NA has proven to have a strong antioxidant capacity by stabilizing free radicals and inhibiting lipid peroxidation [14]. Previous studies have reported NA supplementation to extenders in the freezing of pig [15] and stallion [16] semen, and that NA protects the post-thaw spermatozoa plasma membrane integrity and increases total antioxidant activity by reducing lipid peroxidation [15,16].

Folic acid (FA) catalyzes reactions related to the synthesis of nucleic acids and proteins, but also protects cell membranes and DNA against free radical damage due to its strong lipid peroxidation inhibitory activity [17,18]. A study evaluating NA and FA in human semen freezability reported that the preservation of post-thaw DNA integrity in the NA/FA combination group was due to the synergistic effect of both antioxidants, where metabolically inactive FA was activated by NA [19]. In another study conducted in human semen, post-thaw spermatozoa viability, plasma membrane integrity, and chromatin quality rates were found to be highest in either the FA or the NA/ FA combination group [20]. Based on an exhaustive search of the available literature, no previous studies have evaluated the combined effects of NA and FA supplementation in extenders for ram semen cryopreservation.

Given the limited predictive value of traditional semen parameters such as motility, concentration, and morphology, there is a growing need to complement these assessments with molecular-level analyses to better understand and predict male fertility potential [21,22]. Therefore, an attempt is made to develop molecular biomarkers in spermatozoa that will more reliably reflect sperm fertility. In the present study, in addition to traditional spermatological parameters, the effects of NA and FA combination on the freezability of ram semen were evaluated, especially at the gene level.

Guanine is especially susceptible to ROS-induced oxidative damage due to its low redox potential, leading to the formation of lesions such as 8-oxoguanine (8-oxoG) [23,24]. These are primarily repaired via the base excision repair (BER) pathway, initiated by 8-oxoguanine DNA glycosylase 1 (*OGG1*), which identifies and removes 8-oxoG lesions [25,26].

Localized in the mitochondria, ROS modulator 1 (*ROMO1*) is a major gene responsible for triggering mitochondrial ROS production during oxidative stress [27]. Besides promoting mitochondrial ROS production, *ROMO1* is also essential for preserving mitochondrial membrane potential [28].

*OGG1* and *ROMO1* were selected because they represent two complementary molecular aspects of cryopreservation-induced oxidative stress in spermatozoa. *OGG1* is involved in the recognition and repair of oxidative DNA lesions, whereas *ROMO1* is closely associated with mitochondrial ROS production and mitochondrial function. Therefore, evaluating these genes may provide insight into both oxidative DNA damage responses and mitochondrial redox alterations in cryopreserved ram spermatozoa [25,26,27,28].

The present study aimed to investigate the effects of different combinations of nicotinic acid NA and FA on post-thaw ram semen quality, with particular emphasis on spermatological characteristics and the expression and methylation profiles of the ROS-related genes *OGG1* and *ROMO1*.

## 2. Results

The evaluation of fresh ram semen at the beginning of the study revealed mean values of 80.33% for total motility, 93.67% for Hypoosmotic Swelling Test (HOST), 14.33% for acrosomal membrane damage as evaluated by the FITC-conjugated Pisum sativum agglutinin assay (FITC-PSA), 6.33% for low mitochondrial membrane function (JC-1), and 4.33% for DNA damage, as evaluated by the terminal deoxynucleotidyl transferase dUTP nick-end labeling assay (TUNEL), along with antioxidant levels of 0.71 nmol/mL for Malondialdehyde (MDA) and 13.40 U/mL for Total Antioxidant Capacity (TAC). A significant decrease in each of these values was observed following the freeze–thaw process (*p* < 0.05).

### 2.1. Sperm Motility and Kinematic Parameters

No significant differences in total motility (TM) were observed among the groups; however, the highest numerical value was recorded in the NA10FA50 group (53.34%), whereas the lowest was observed in the control group (43.44%). It was determined that the motility value of the NA20FA50 group was lower than that of the NA10 or NA10FA50 groups (Table 1).

Regarding the Amplitude of Lateral Head Displacement (ALH), Distance Average Path (DAP), Distance Curved Line (DCL), Curvilinear Velocity (VCL) and Straight-Line Velocity (VSL) ratios, the control group was significantly higher than the NA20FA50 group, but only numerically higher than the NA10 and NA10FA50 groups. The Beat Cross Frequency (BCF) and Average Path Velocity (VAP) values of the control group were also similar to the NA10FA50 group (*p* > 0.05), but statistically higher than the NA10 and NA20FA50 groups (*p* < 0.05). Only the NA10 group was found to be statistically higher than the control group in terms of Linearity (LIN), Straightness (STR) and Wobble (WOB) values (*p* < 0.05). In terms of Distance Straight Line (DSL), only the NA20FA50 group had the lowest value, which was significantly lower than the control and NA10FA50 (*p* < 0.05) (Table 2).

### 2.2. Plasma Membrane Integrity

There were no statistical differences in terms of plasma membrane integrity among the groups, although numerically the highest plasma membrane integrity value was found in the NA10FA50 group (*p* > 0.05) (Table 1).

### 2.3. Acrosomal Membrane Integrity

Although the lowest acrosomal damage was obtained in the NA10FA50 group, it was only statistically different from the control group (*p* < 0.05) (Table 1).

### 2.4. Mitochondrial Membrane Potential

Mitochondrial membrane damage was highest in the control group and significantly differed from all antioxidant groups (*p* < 0.05) (Table 1).

### 2.5. DNA Fragmentation

There was no statistical difference in terms of DNA integrity among the groups (*p* > 0.05) (Table 1).

### 2.6. Malondialdehyde and Total Antioxidant Capacity

No statistical difference was found between the groups in terms of MDA and TAC values (*p* > 0.05) (Table 3).

### 2.7. Expression and Methylation Levels of the Ovine OGG1 and ROMO1 Genes

**Figure 1:**
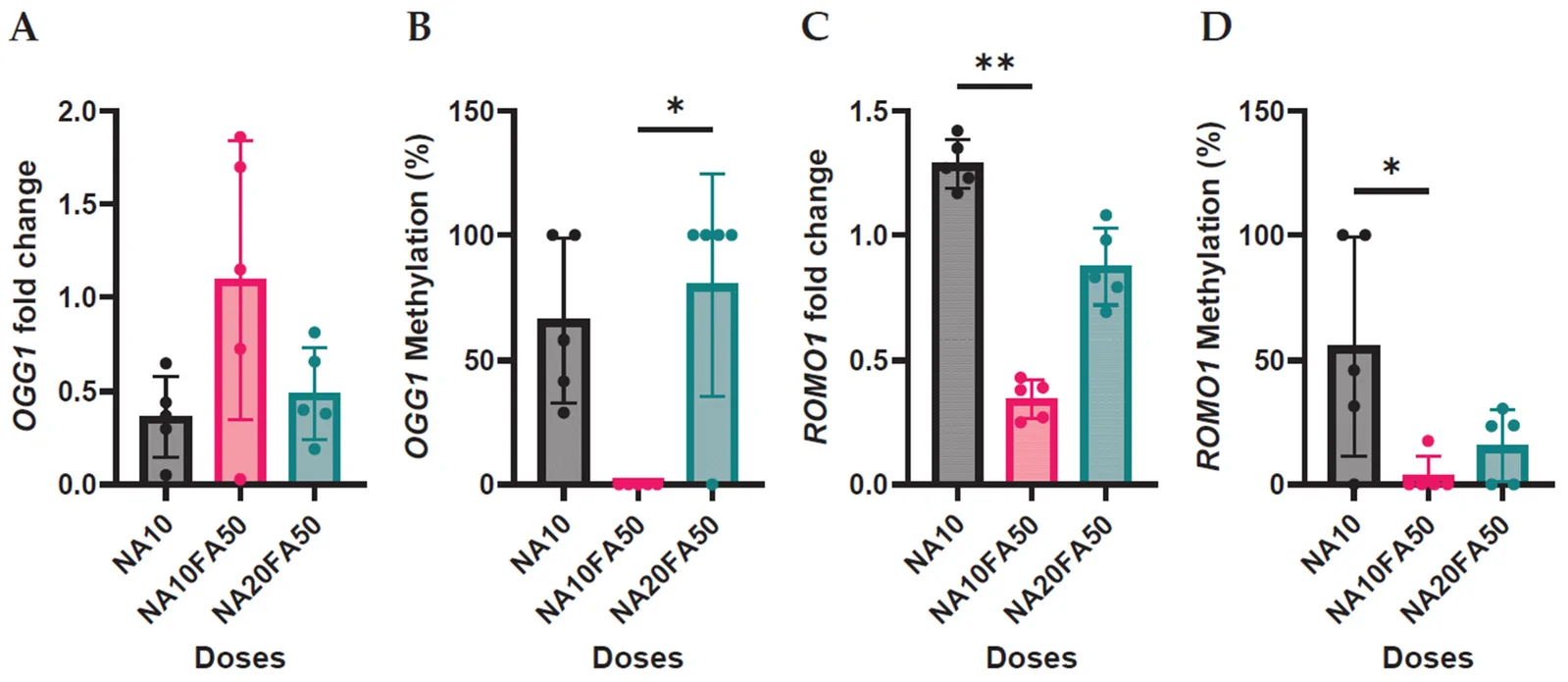
Effects of different doses of Nicotinic acid (NA) and different doses of Nicotinic acid/Folic acid combination (NAFA) on sperm *OGG1* and *ROMO1* gene regulation: (**A**) impact of NA/FA combination on *OGG1* gene expression, (**B**) impact of NA/FA combination on *OGG1* gene methylation, (**C**) impact of NA/FA combination on *ROMO1* gene expression, (**D**) impact of NA/FA combination on *ROMO1* gene methylation. The expression levels of the target genes were normalized to *ACTB* as a reference gene. Fold changes in gene expression were calculated using the 2^−ΔΔCt^ method. (* *p* < 0.05, ** *p* < 0.01). Each dot represents one independent biological replicate (*n* = 5). Bars represent the mean ± standard deviation.

To investigate the epigenetic regulation and expression dynamics of *OGG1* and *ROMO1* in response to combined exposures, their mRNA fold changes, and promoter methylation percentages were quantified, as shown in Figure 1. The *OGG1* expression was higher in the NA10FA50 group, whereas a marked decrease in *OGG1* expression was observed in the NA20FA50 group (Figure 1A). Correspondingly, *OGG1* promoter methylation was elevated in NA20FA50 compared to NA10FA50 (*p* < 0.05) (Figure 1B), suggesting a potential inverse relationship between methylation and gene expression. This epigenetic silencing effect is consistent with established models of transcriptional repression via DNA methylation.

For *ROMO1*, a different pattern emerged. Expression was lowest in NA10FA50 and remarkably increased in NA20FA50 (Figure 1C). However, the highest *ROMO1* expression was observed for the NA10 (*p* < 0.01). Surprisingly, *ROMO1* promoter methylation also increased progressively for this condition (*p* < 0.05) (Figure 1D), suggesting a disconnect between methylation and transcriptional repression in this gene, potentially due to non-functional methylation sites or compensatory transcriptional regulation. It is important to note that expression and methylation patterns exhibit an inverse relationship in the NA/FA combinations, similar to that observed for *OGG1*. Collectively, these findings highlight a dose-dependent epigenetic suppression of *OGG1*, while *ROMO1* shows methylation-insensitive upregulation of expression under combined exposures.

## 3. Discussion

ROS are involved in regulating essential sperm functions such as capacitation, hyperactivation, acrosome reaction, and spermatozoa–oocyte fusion [29]. However, excessive ROS production impairs plasma membrane integrity, reducing motility, metabolism, and viability [30]. To improve frozen-thawed semen quality, antioxidant supplementation in extenders has been widely reported [7,8,9]. Studies have shown that antioxidant substances can exhibit various effects on sperm cryopreservation, both when used alone and in combination with different antioxidants [31]. While these effects may be synergistic in some cases, other studies have reported that the combined use of different compounds does not always produce the same effect [32]. Furthermore, it has been emphasized that the biological effects of many antioxidants are dose-dependent, and applications outside the optimal concentration range may fail to provide the expected benefits [33,34,35,36]. Here, the effects of nicotinic and folic acid on the freezability of ram semen were specifically evaluated at the gene level.

Consistent with the findings of Peris et al. [37], Mahmuda et al. [38], Ustuner et al. [39], and Banday et al. [40], the current study confirms that cryopreservation significantly impairs fresh sperm quality parameters (*p* < 0.05), with the most pronounced negative effects observed in the control group.

Sperm motility is crucial for enabling a spermatozoon to access the fertilization region and penetrate both the oocyte’s cumulus oophorus complex and zona pellucida [1]. According to the data obtained in the present study, no statistically significant differences in TM evaluated by Computer-Assisted Sperm Analysis (CASA) were observed among the groups (*p* > 0.05). However, the highest numerical mean TM value was observed in the NA10FA50 group (53.34%), whereas the lowest numerical mean TM value was observed in the control group (43.44%). In the study conducted by Rarani et al. [19] with NA and FA combinations in human semen, the NA (10 mM) group was not different from the control group and the NA10FA50 group had the highest post-thaw motility, which is consistent with the presented study. The post-thaw TM values obtained via CASA in the present study were consistent with the previously reported objective motility range (41.89–62%) for cryopreserved ram semen [9,41,42]. Also, earlier reports based on subjective evaluations indicate post-thaw motility values ranging from 31.13% to 68.00% [39,43,44,45]. The present study applies an objective and standardized CASA system to eliminate subjectivity and evaluator-dependent variability inherent in motility assessments. The motility values obtained using CASA are consistent with the objective reference ranges reported in the scientific literature, supporting the reliability of our findings.

Increased ALH value indicates lower sperm quality, which can disrupt the progressive movement of spermatozoa [46]. In fact, it was stated by Santiago–Moreno et al. [47] that an increase in ALH is associated with the onset of early capacitation. Considering ALH ratios, it was determined that the control group was numerically higher than all groups. In line with this information about ALH, it can be considered that the control group underwent an early capacitation process compared to the antioxidant groups. The BCF is positively correlated with fertility to identify changes in the flagellar rhythm pattern [48]. The highest BCF value (38.04) was observed in the NA10FA50 group.

When the linearity parameters STR, LIN and WOB were analyzed, only the NA10 group was statistically higher than the control group, NA10FA50 and NA20FA50 were found numerically higher. The elevated linearity parameters observed in the NA10 group may be attributed to the role of NA in regulating mitochondrial Nicotinamide Adenine Dinucleotide (NAD^+^) and Nicotinamide Adenine Dinucleotide Phosphate (NADP^+^) levels, which are essential cofactors in the Tricarboxylic Acid (TCA) cycle [49,50]. By enhancing mitochondrial oxidative metabolism and ATP production, nicotinic acid likely supports the energy demands of sperm motility, thereby increasing kinematic activity. In general, higher VCL values with ALH are undesirable as they indicate hyper-activated spermatozoa [48]. It was observed that higher ALH and velocity parameters (VCL, VSL, VAP) of the control group, which were compared to the antioxidant groups, showed early hyperactivation, and the antioxidant groups protected the spermatozoa from this undesirable situation.

Sperm kinematic parameters such as VCL, VSL, VAP, LIN, STR and WOB are associated with fertility [51,52]. In contrast, Assunção et al. [53] noted that higher values obtained in kinetic motion parameters are not always associated with a higher fertilization rate. Semen parameters determined by CASA depend on the type of slide/camera used, extender composition, semen processing method, operator’s choice of microscopic area for analysis (frame rate), as well as CASA systems of different brands and models, causing the specified variations to occur [54].

Some researchers claim that antioxidant activity is dose-dependent, and excessive doses of antioxidants cause apoptosis with pro-oxidant effect [55,56]. Therefore, it is thought that the NA20FA50 group suppresses motility. It was determined that the NA10 and NA10FA50 groups showed similar effects on post-thaw total motility, but when evaluated using kinematic parameters, the combination group was found to be more advantageous.

Folic acid is significantly soluble in water; it has been reported to have strong lipid peroxidation inhibitory activity, thus protecting biological compounds such as cell membranes and DNA against free radical damage [18]. Nicotinic acid regulates the NAD and NADP coenzymes in mitochondria, which are involved in the TCA cycle and support cellular energy production [50]. The preservation of plasma membrane functional integrity is essential for the spermatozoon to interact with its external environment, to enable spermatozoa–oocyte fusion during its residence in the female reproductive tract, and to initiate the acrosome reaction [57]. The obtained post-thaw plasma membrane functional integrity values ranged from 35.21% to 71.70%, which is consistent with the findings reported in previous studies [10,39,40,41,58]. Post-thaw plasma membrane integrity did not differ statistically among the groups, but the highest numerical value was observed in NA10FA50 (73.00%). In a study on human semen, Hashemi et al. reported that they found a higher level of plasma membrane integrity in the 10 mM NA 50 nM FA combination group compared to other groups, similar to the current study [20].

In the study conducted by Lee et al. [15] on boar semen, the administration of 10 mM NA resulted in a statistically significant improvement in sperm viability compared to the control group; however, this effect was not reflected in plasma membrane integrity, a finding that aligns with the current study. It was also reported that increasing doses of NA had a negative effect on sperm viability. Consistent with this, Lee et al. [15] in the 20 nM dose NA group, similar to our current study, showed that the depressive effect was detected in NA20FA50, which had the lowest plasma membrane integrity among the groups.

Cryopreservation causes several changes in the spermatozoa acrosome and calcium channels, termed cryo-capacitation [59]. While discussing the kinematic parameters, it was noted that the control group underwent an earlier capacitation process than the antioxidant groups. In parallel, it was observed that acrosome damage was higher in the control group. In terms of acrosomal integrity, the NA10FA50 group was superior to the other groups but only statistically different from the control group (*p* < 0.05). The post-thaw acrosome defect data obtained in this study are consistent with previous reports indicating acrosome damage rates ranging from 35% to 69% due to the freeze–thaw process [39,58,60,61].

In cryopreservation, decreased mitochondrial activity due to increased mitochondrial membrane permeability leads to the release of pro-apoptotic factors from the mitochondria, negatively affecting sperm viability [62]. Therefore, MMP, an indicator of mitochondrial activity, has been recognized as a predictor of sperm quality reflecting the energy status and respiratory chain activity in the mitochondria [63]. Nicotinic acid has been reported to support mitochondrial activity by protecting spermatozoa against ROS-mediated MMP disruption through its antioxidant properties [64]. In this study, antioxidant groups were found to protect the MMP against the negative effects of cryopreservation compared to the control group (*p* < 0.05). In particular, the NA/FA combination groups showed an improved mitochondrial membrane potential compared to the NA10 group. Also, based on previous studies [65,66], researchers found that FA is not metabolically active and NA increases cell energy levels by producing the coenzyme NADP, which can potentiate the antioxidative activity of FA [19].

Although antioxidant supplementation exerted beneficial effects on MMP, no significant differences in DNA fragmentation were detected among the groups. Smith et al. [67] reported that the early stages of DNA damage could not be effectively detected by the TUNEL staining method and that TUNEL-positive signals were primarily observed after 24–48 h of incubation at 37 °C. In this context, the lack of a statistically significant difference between the groups in terms of DNA damage in thawed ram spermatozoa observed in our study may be attributed to the fact that the samples were evaluated in the post-thaw stage at 0 h without any additional incubation period according to our experimental design. The absence of a statistically significant difference in DNA fragmentation among the groups suggests that the TUNEL method lacks sufficient sensitivity for this purpose. The current study’s findings are supported by similar outcomes from other studies that also used the TUNEL method to evaluate spermatozoa DNA fragmentation [43,44,68,69].

In the current study, when post-thaw MDA and TAC levels were examined, no significant differences were observed. However, Soleimanzadeh et al. [10] reported that 50 and 100 μM doses of caffeic acid reduced MDA levels and increased TAC levels in buffalo. In a study comparing the addition of various antioxidants to the extenders used in the cryopreservation of ram semen, Banday et al. [40] and Inanc et al. [70] reported that, similar to our current study, there was no statistical difference in post-thaw MDA levels when the control group and antioxidant groups were compared. In a study by Bucak et al. [71] comparing the effects of different antioxidants on the cryopreservation of goat semen, hypotaurine is known to help preserve post-thaw motility and plasma membrane integrity, the interpretation of MDA and TAC values was found to be consistent with the findings of our study. They reported that the increase in MDA and the low TAC levels were due to changes in the cryopreservation protocol and experimental design, extender formulas, dilution rate, antioxidant dosages and animal species or breed variability.

This study analyzed the expression levels of *ROMO1* and *OGG1* to validate data from MDA, TAC, mitochondrial membrane potential, and TUNEL assays. Previous studies have reported reduced *OGG1* and *ROMO1* expression in cryopreserved canine semen and negative associations between the expression of these genes and sperm quality parameters [72,73]. However, information regarding the expression and methylation status of *OGG1* and *ROMO1* in relation to male reproductive function remains limited. Further molecular studies are therefore needed to elucidate the effects of cryopreservation on sperm function in both humans and animals.

*OGG1* plays a central role in DNA base excision repair and has been implicated in reproductive physiology [74]. The quantification of *OGG1* mRNA and promoter methylation revealed dose-dependent changes in gene expression and methylation. Specifically, *OGG1* expression peaked under the NA10FA50 treatment but was substantially repressed in the NA20FA50 group. This reduction was paralleled by increased promoter methylation, suggesting epigenetic silencing through DNA hypermethylation at higher exposure levels. These findings align with established epigenetic models where promoter methylation suppresses gene transcription, reinforcing the idea that *OGG1* is epigenetically modulated in a dose-sensitive manner [75,76,77]. It is important to note that group comparisons revealed a striking pattern between *OGG1* gene expression and promoter methylation across the treatment groups; however, Spearman correlation analysis did not detect a statistically significant association between these variables (ρ = −0.271, *p* = 0.329) as shown in Figure S1.

The biological relevance of these remarkable findings is underscored by the functional role of *OGG1* in protecting genomic integrity. Its suppression may compromise DNA repair capacity in spermatozoa during cryopreservation, potentially affecting fertility outcomes [74,78]. Thus, evaluating *OGG1* regulation at both expression and methylation levels provides a more comprehensive understanding of how antioxidants may influence male reproductive fitness in rams. In the present study, NA/FA supplementation was associated with alterations in both *OGG1* expression and promoter methylation. The inverse pattern observed between these parameters suggests a potential relationship between promoter methylation status and *OGG1* transcription; however, the current experimental design does not permit establishment of a causal relationship. This outcome reinforces the notion that changes in gene expression are functionally associated with methylation patterns. Accordingly, the changes in gene expression levels observed here are consistent with current scientific understanding and may shed light on RNA and transcription activity in spermatozoa [79,80,81]. The observed variations in the expression of ROS-regulating genes in this study are of notable importance.

In contrast, *ROMO1*, a gene associated with mitochondrial ROS generation, showed a different regulatory pattern. *ROMO1* has been implicated in multiple biological contexts, including sperm function in buffalo bulls [82], immune modulation in prostate cancer [83], and mitochondrial metabolism and ROS sensing [84]. In our study, *ROMO1* expression was lowest in NA10FA50 and significantly elevated in the NA10 group, with the highest levels observed in the NA10 group. Interestingly, promoter methylation of *ROMO1* also increased progressively, indicating a lack of classical inverse correlation between methylation and transcriptional repression. This decoupling suggests that *ROMO1* expression may be regulated through alternative mechanisms, such as transcription factor binding and post-transcriptional modulation, or that methylation occurs in regions not critical for gene silencing [85,86]. It also raises the possibility that *ROMO1* methylation may play a context-dependent or compensatory role in response to oxidative stress stimuli induced by cryopreservation process. Studies have shown that supplementing extenders with antioxidants during sperm cryopreservation across various species reduces post-thaw *ROMO1* gene expression, and that *ROMO1* expression is negatively correlated with high MMP [72,73,87,88,89]. The negative correlation noted between the NA10 and NA10FA50 groups was also observed in the current study. Low MMP was observed in the NA10 group with the highest *ROMO1* expression level, while high mitochondrial membrane potential was observed in the NA10FA50 group with the lowest *ROMO1* expression level.

Importantly, *OGG1* exhibited an inverse association between promoter methylation and gene expression, consistent with a canonical methylation-associated repression pattern, whereas ROMO1 did not show a comparable relationship. These contrasting patterns highlight the complexity and gene-specific nature of the associations between promoter methylation and transcription [75,90]. Nevertheless, the present data are correlative and do not establish promoter methylation as the direct regulatory mechanism underlying the observed changes in gene expression. Notably, although the group means suggested an inverse pattern between *OGG1* expression and promoter methylation, this trend was not confirmed by Spearman correlation analysis (Figure S1). Therefore, the observed changes should be interpreted as treatment-associated group differences rather than evidence of a direct relationship between *OGG1* expression and promoter methylation. These differential patterns emphasize the gene-specific nature of epigenetic regulation [75,90,91]. Nevertheless, in the NA/FA combination groups, expression and methylation profiles showed an inverse trend similar to that of *OGG1*, supporting the general notion that exposure concentration influences epigenetic programming.

Taken together, our findings demonstrate the value of integrating gene expression and promoter methylation analyses when investigating the molecular responses associated with cryopreservation additives. The observed relationships between methylation and expression provide a basis for further investigation of potential epigenetic involvement in the regulation of *OGG1* and *ROMO1*. However, mechanistic studies are required to determine whether the observed methylation changes directly contribute to altered gene expression and whether these molecular alterations have functional consequences for sperm fertility. Further research is warranted to elucidate whether these molecular alterations have functional consequences on fertility traits and to assess their reversibility or long-term heritability.

## 4. Materials and Methods

This study used five Merino rams aged 2–4 years with confirmed reproductive capability. The rams were maintained by the Faculty of Veterinary Medicine at Bursa Uludag University (Northwestern Anatolia, Turkey) with unrestricted access to fresh water, maize, and alfalfa hay. All experimental procedures and evaluation methods were approved by the Scientific Ethical Committee of Bursa Uludag University (approval no. 2021-09/02).

### 4.1. Preparation of Semen Extenders and Chemical Composition

Tris-based extenders were prepared for use in a two-step dilution method. Extender A contained 223.7 mmol Tris, 66.6 mmol citric acid, 55.5 mmol fructose, 3 g/L dihydrostreptomycin, 4 g/L penicillin G, and 20% (*v*/*v*) egg yolk. Extender B was formulated by adding 4.03 mmol ethylenediaminetetraacetic acid (EDTA), 100.4 mmol trehalose, and 6% (*v*/*v*) glycerol to the composition of Extender A, based on the method described by Aisen et al. [92]. Unless stated otherwise, all chemicals used throughout the study were obtained from Merck (Darmstadt, German) and Sigma (Burlington, MA, USA).

**Figure 2:**
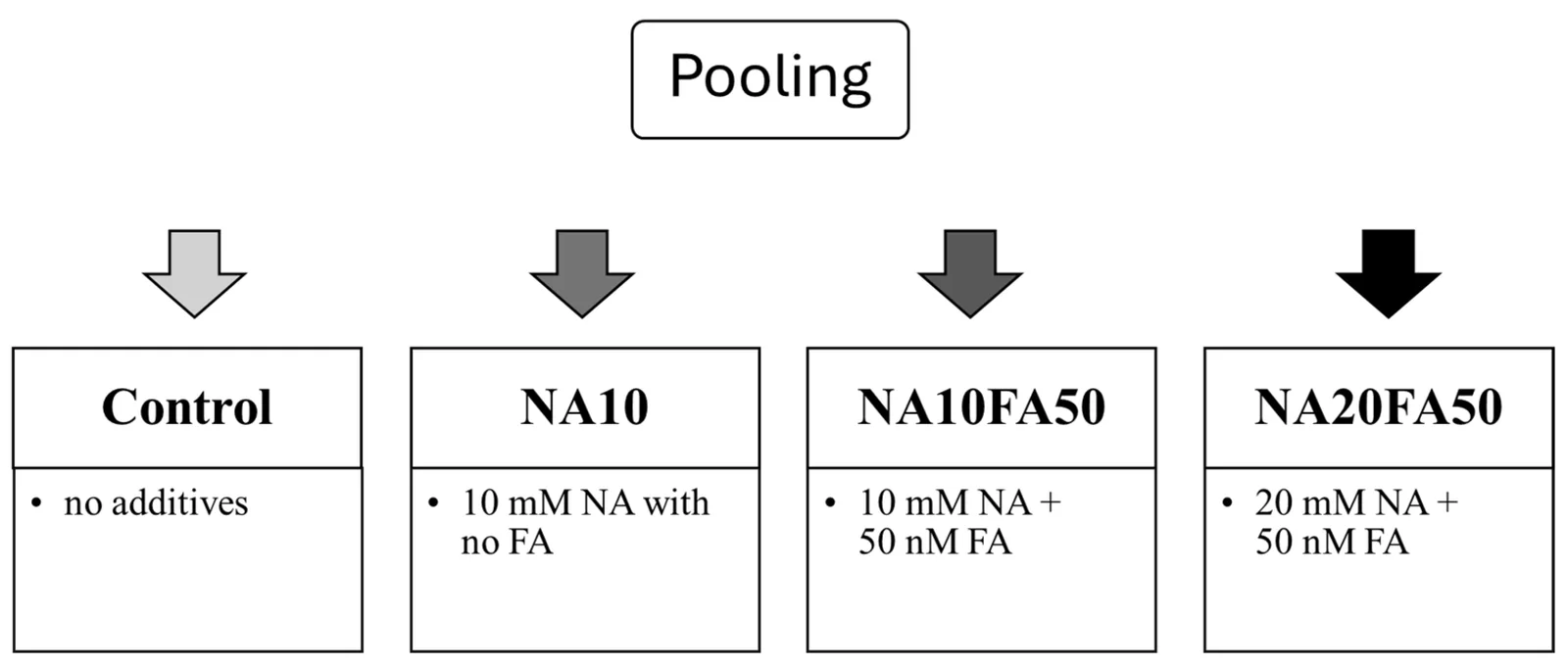
Schematic representation of extender preparation for the Control, 10 mM NA with no FA (NA10), 10 mM NA + 50 nM FA (NA10FA50) and 20 mM NA + 50 nM FA (NA20FA50).

In the experimental design, extenders were supplemented with different concentrations of NA (Sigma-Aldrich—N0761, İstanbul, Türkiye) and FA (Sigma-Aldrich—F8758, İstanbul, Türkiye). Accordingly, the study comprised four groups: Control (no antioxidants), 10 mM NA with no FA (NA10), 10 mM NA + 50 nM FA (NA10FA50) and 20 mM NA + 50 nM FA (NA20FA50), as illustrated in Figure 2.

### 4.2. Collection, Dilution, Freezing, and Thawing of Semen

In the non-breeding season (November–December), semen collection was performed five times on alternate days using an electro-ejaculator for small ruminants (Minitüb GmbH, Tiefenbach, Germany; REF: 11900/0000) as described by Ustuner et al. [39]. The samples were kept in a warm water bath (28–30 °C) during transport to the laboratory, where they were evaluated for mass activity (0–5 scale), motility, and sperm concentration [39]. Only ejaculates with a mass activity of +++ or above, at least 70% motility, and a minimum concentration of 1 × 10^9^ spermatozoa/mL were pooled.

Pooled ejaculates were diluted using a two-step dilution protocol, with the final concentration adjusted to 80 × 10^6^ spermatozoa per straw. Samples were divided into four equal parts, each diluted with Extender a according to the respective group: Control, NA10, NA10FA50, and NA20FA50. The diluted samples were then gradually cooled to 5 °C for one hour. Following the cooling process, a second dilution was performed using Extender B for each group, and the samples were equilibrated at 5 °C for two hours.

After equilibration, semen samples were loaded into 0.25 mL straws and cryopreserved using a Nicool Plus PC freezing system (Air Liquide, Marne-la-Vallée Cedex 3, France). The freezing protocol included an initial cooling of the semen from +5 °C to −8 °C at a rate of 3 °C per minute, followed by a rapid drop from −8 °C to −120 °C at 15 °C per minute using liquid nitrogen vapor. The straws were then stored in liquid nitrogen at −196 °C for at least 1 month. For post-thaw semen evaluation, at least three straws per group were thawed in a 37 °C water bath for 30 s.

### 4.3. Evaluation of Semen

All semen parameters were evaluated at both the native and post-thaw stages, with all assessments consistently performed by the same individual throughout the study.

#### 4.3.1. Evaluation of Sperm Mass Activity, Motility and Kinematic Parameters

Sperm mass activity was assessed subjectively using a phase-contrast microscope (Olympus BX51, Tokyo, Japan) at 100× magnification, equipped with a heated mechanical stage, following the method described by Ustuner et al. [39].

To evaluate sperm motility and kinematic parameters, CASA (Ceros II, IMV Technologies) was used. The parameters measured included TM (%), ALH (μm), BCF (Hz), DAP (μm), DCL (μm), DSL (μm), LIN (VSL/VCL, %), STR (VSL/VAP, %), VAP (μm/s), VCL (μm/s), VSL (μm/s), and WOB (VAP/VCL, %). According to the manufacturer’s instructions, semen samples were diluted using a Tris-based extender, and 3 µL of the diluted sample was placed on a pre-warmed (37 °C) analysis slide. In accordance with the recommendations of Dayanikli et al. [93], a minimum of eight fields or 400 spermatozoa were examined during each CASA analysis to ensure reliable data.

#### 4.3.2. Evaluation of Sperm Plasma Membrane Functional Integrity via the Hypoosmotic Swelling Test

According to the method described by Ustuner et al. [39], functional membrane integrity was assessed by mixing 10 µL of semen with 100 µL of a 100 mOsM hypoosmotic solution, followed by incubation at 37 °C for 1 h. Following incubation, 200 spermatozoa were examined using phase-contrast microscopy to detect any coiled tails.

#### 4.3.3. Evaluation of Sperm Acrosome Integrity via FITC Conjugated Pisum Sativum Agglutinin Assay

**Figure 3:**
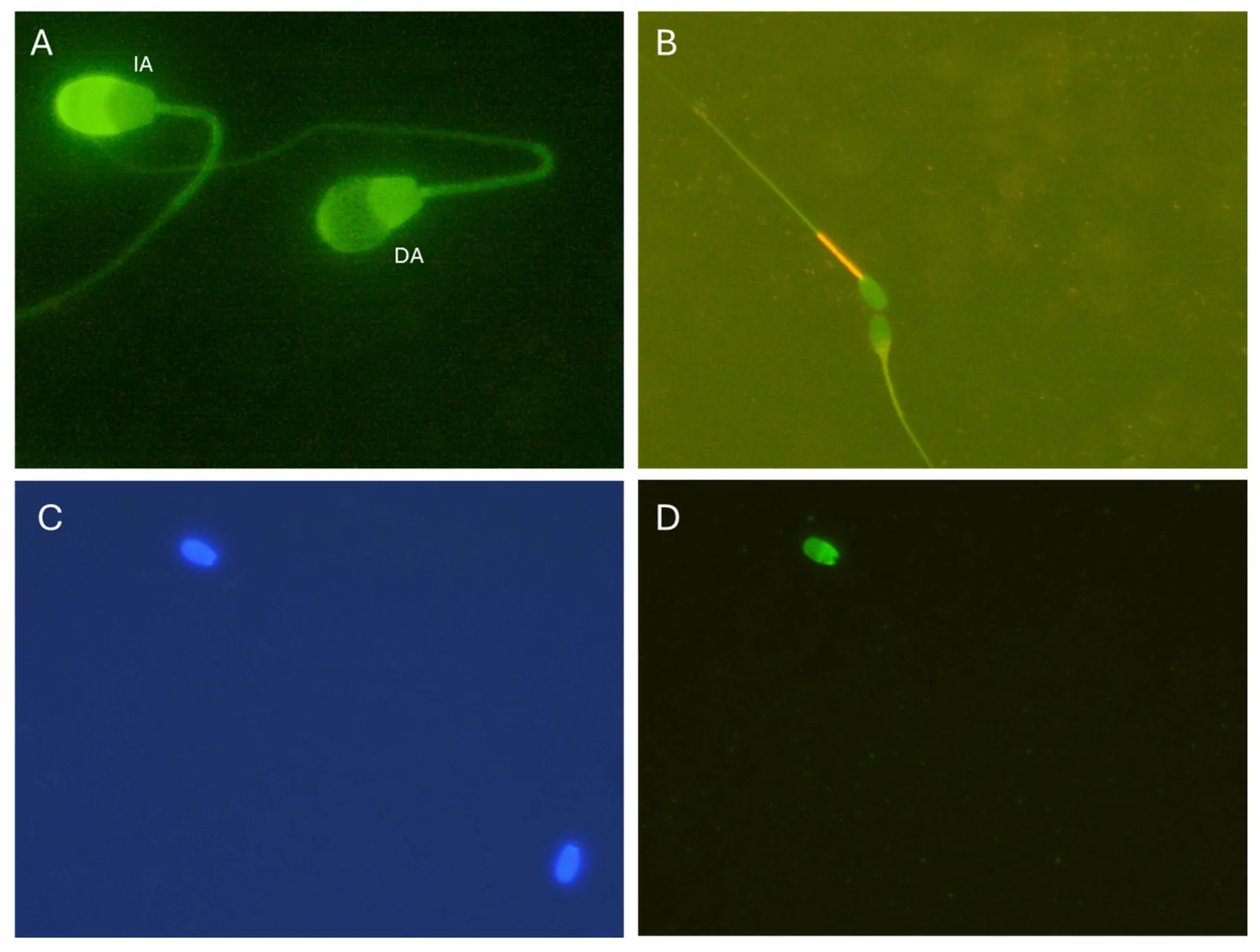
Representative patterns of post-thaw ram semen as observed with FITC-PSA, JC-1 and TUNEL staining, respectively. (**A**): FITC-PSA staining for acrosomal membrane integrity evaluation (×100). DA: Defected Acrosome, IA: Intact Acrosome. (**B**): JC-1 staining for mitochondrial membrane potential assessment. Yellow/orange and green fluorescence associated with the midpiece of spermatozoa indicated a high and a low mitochondrial membrane potential, respectively (×40). (**C**,**D**): Evaluation of sperm DNA fragmentation using TUNEL assay. (**C**): DNA in both normal and spermatozoa with fragmented DNA in DAPI (blue) and (**D**): DNA-fragmented spermatozoa stained green as TUNEL (+) cells, respectively (×40).

The assessment of acrosomal integrity was performed as described by Nur et al. [2]. Slides were stained and examined under a fluorescence microscope, and at least 200 spermatozoa were analyzed (Figure 3).

#### 4.3.4. Evaluation of Sperm DNA Integrity via Terminal-Deoxynucleotidyl-Transferase-Mediated-dUTP Nick-End Labeling Assay

The TUNEL (Terminal-deoxynucleotidyl-transferase-mediated-dUTP nick-end labeling) technique, slightly modified according to Ustuner et al. (2016), was used to examine DNA integrity in spermatozoa [39]. Following the labeling procedure, slides were analyzed under a fluorescence microscope (Olympus BX 51-TF), and a minimum of 100 spermatozoa were counted to determine the percentage of TUNEL (+) spermatozoa, which indicates spermatozoa with damaged DNA (Figure 3).

#### 4.3.5. Evaluation of Sperm Mitochondrial Membrane Potential via 5,5′, 6,6′-Tetrachloro-1, 1′, 3,3′-Tetraethyl-Benzimidazole Carbocyanine Iodide

The evaluation of spermatozoa mitochondrial membrane potential was carried out using the JC-1 (5,5′, 6,6′-tetrachloro-1, 1′, 3,3′-tetraethyl- benzimidazole carbocyanine iodide) staining protocol based on the method by Inanc et al. [94]. Each sample was analyzed under a fluorescence microscope (Olympus BX 51-TF), with at least 100 spermatozoa examined. JC-1 dye exhibits a reversible change from green to orange fluorescence in response to elevated mitochondrial membrane potential. Spermatozoa showing yellow–orange fluorescence in the midpiece region are interpreted as high mitochondrial activity (Figure 3).

#### 4.3.6. Antioxidant Properties

##### Quantification of Lipid Peroxidation via Malondialdehyde ELISA Assay

Spermatozoa lipid peroxidation was assessed by measuring malondialdehyde (MDA) concentration, a key oxidative stress marker, using a microplate reader (BioTek Epoch, Winooski, VT, USA) and a commercial Enzyme-Linked Immunosorbent Assay (ELISA) kit (BT LAB, Sheep Malondialdehyde, Cat. No. E0039Sh, Shanghai, China). Sperm samples were initially prepared in accordance with the manufacturer’s instructions and loaded into microplate wells pre-coated with MDA-specific antibodies. A biotin-conjugated MDA antibody was then added to bind the MDA present in the samples. Following this, Streptavidin-HRP was introduced to interact with the biotin-labeled antibodies. The microplate was incubated at 37 °C for one hour to allow the reactions to proceed. After incubation, any unbound Streptavidin-Horseradish Peroxidase (HRP) was removed by washing. A substrate solution was then applied, producing a colorimetric change directly proportional to the MDA concentration. The reaction was stopped by adding an acidic stop solution, and absorbance was measured at 450 nm. The results are shown in nmol/mL, after using a standard curve to convert optical density (OD) to MDA concentration.

##### Quantification of Total Antioxidant Capacity via Colorimetric Assay

Total antioxidant capacity (TAC) of the sperm samples was evaluated using a colorimetric assay kit (Elabscience Biotechnology Inc., Houston, TX, USA) in accordance with the manufacturer’s protocol. Following the reaction, OD was measured spectrophotometrically at 520 nm, and TAC levels were calculated using the formula provided with the kit; results were expressed in U/mL.

### 4.4. Genetic Analysis

#### 4.4.1. Nucleic Acid Isolation

Extraction of both RNA and DNA from the sperm samples was undertaken to provide the foundation for the gene expression and methylation analyses. Total RNA was isolated from frozen samples using TRIpure Isolation Reagent (Roche Life Science, Penzberg, Germany), following the manufacturer’s protocol with slight modifications. For DNA extraction, the High Pure Polymerase Chain Reaction (PCR) Template Preparation Kit (Roche Diagnostics GmbH, Mannheim, Germany) was used according to the supplier’s instructions. The RNA samples were subsequently used for gene expression studies, while the purified DNA was employed for methylation analysis.

#### 4.4.2. Evaluation of Ovine OGG1 and ROMO1 Gene Expression Alterations

Following RNA isolation, cDNA was synthesized using the RevertAid RT Reverse Transcription Kit (Thermo Fisher Scientific) with 1000 ng of RNA. Gene expression analysis was performed via real-time PCR using the LightCycler 480 II system (Roche) and SYBR Green I Master Mix. Primers for *OGG1*, *ROMO1*, and the reference genes ACTB and GAPDH were designed specifically for Ovis aries based on NCBI and ENSEMBL database sequences, and their specificity was confirmed using BLAST v2.16.0 (NCBI; https://blast.ncbi.nlm.nih.gov/) (Table 4). Quantification was carried out using the LightCycler 480 Relative Quantification Software, and expression levels were normalized to Actin Beta (*ACTB*) and Glyceraldehyde-3-Phosphate Dehydrogenase (*GAPDH*). Fold changes were calculated using the 2^–ΔΔCt^ method [95]. After evaluating reference gene stability, *ACTB* was selected as the most stable and used for final normalization.

Primer amplification efficiencies were determined using standard curves generated from a [five]-point serial dilution of pooled cDNA. Each dilution was analyzed in duplicate. Amplification efficiency was calculated from the slope of the standard curve using the equation E = (10 − 1/slope − 1) × 100. Primer pairs were considered acceptable when efficiencies ranged from 90% to 110% and coefficients of determination (R^2^) ≥ 0.98. The corresponding amplicon sizes were 95 bp for *OGG1*, 97 bp for *ROMO1*, 100 bp for *ACTB*, and 123 bp for *GAPDH*. Primer specificity was initially assessed by BLAST and subsequently confirmed by a single peak in the melting-curve analysis. No amplification was observed in no-template controls or no-reverse-transcription controls. Reference-gene stability was assessed using RefFinder, incorporating the comparative ΔCt method, BestKeeper, NormFinder, and geNorm. *ACTB* showed the highest overall stability and was therefore selected for normalization.

#### 4.4.3. Evaluation of DNA Methylation

To evaluate DNA methylation, genomic DNA was bisulfite-converted using the EZ DNA Methylation-Gold Kit (Zymo Research, Irvine, CA, USA) prior to real-time PCR analysis. Primers targeting CpG-rich regions within the *OGG1* and *ROMO1* genes were designed using MethPrimer software (https://www.methprimer.com), based on sequences from the NCBI and ENSEMBL databases. Both methylation-specific and unmethylation-specific primers (Table 5) were used to amplify bisulfite-converted DNA on the LightCycler 480 II system (Roche), employing SYBR Green I Master Mix. Quantitative methylation-specific PCR (qMSP) was performed using methylated- and unmethylated-specific primer sets on the LightCycler® 480 Real-Time PCR System (Roche Diagnostics). The methylation percentage of each sample was calculated using the following formula: Methylation (%) = 100/[1 + 2^(CtM − CtU)], where CtM and CtU represent the threshold cycle (Ct) values obtained with the methylated- and unmethylated-specific primer sets, respectively. Methylated and unmethylated control DNA were used to verify assay performance, while melting curve (Tm calling) analysis was performed for all reactions to evaluate the melting profiles of the amplified products.

Methylation profiles were analyzed using the Tm Calling Mode of the LightCycler 480 software, with melting temperature shifts distinguishing methylated from unmethylated alleles. A fully methylated control DNA was included for validation. Successful conversion and assay performance were indirectly supported by the expected amplification profiles obtained with methylation-specific and unmethylation-specific primer sets, the use of fully methylated control DNA, and the clear discrimination of melting peaks during melting curve analysis.

RT-qPCR and methylation-specific real-time PCR analyses were performed using samples obtained from five independent biological replicates. Each biological replicate was analyzed in duplicate, and the mean Ct value of the technical duplicates was used for subsequent analyses.

### 4.5. Statistical Analysis

Spermatological parameters and antioxidant properties were analyzed using SPSS version 26 for Windows (SPSS Inc., Chicago, IL, USA). The Shapiro–Wilk test was applied to assess the normality of the data, while Levene’s test was used to determine the homogeneity of variances. For datasets meeting both assumptions, one-way ANOVA was conducted, followed by Tukey’s post hoc test for pairwise group comparisons. Parameters that did not conform to normal distribution were evaluated using the Kruskal–Wallis and Mann–Whitney U tests for group and pairwise comparisons, respectively. For Kruskal–Wallis analyses, effect sizes were calculated using epsilon squared (ε^2^), according to the formula ε^2^ = (H − k + 1)/(*n* − k), where H is the Kruskal–Wallis statistic, k is the number of groups, and n is the total number of observations. Effect size measures were calculated and are provided in Supplementary Table S1.

Statistical analyses of gene expression and methylation data were performed using GraphPad Prism 9 (GraphPad Software, San Diego, CA, USA). Data normality was assessed using the Anderson–Darling test. As the data did not meet the assumptions of normality, group comparisons were conducted using the Kruskal–Wallis test, followed by Dunn’s post hoc test for multiple comparisons. A *p*-value < 0.05 was considered statistically significant, whereas *p*-values ≥ 0.05 were considered non-significant.

## 5. Conclusions

To our knowledge, this study is the first to investigate the association between DNA methylation patterns and the expression of the *OGG1* and *ROMO1* genes following supplementation with nicotinic acid and folic acid during ram semen cryopreservation. Supplementation of the extender with 10 mM nicotinic acid alone or in combination with 10 mM folic acid was associated with improved post-thaw sperm quality, particularly with respect to acrosomal integrity, mitochondrial membrane potential, and selected molecular markers. These findings suggest that nicotinic acid and folic acid may contribute to improving post-thaw sperm quality, possibly through modulation of oxidative stress-related gene expression and epigenetic status. However, the observed associations between DNA methylation and gene expression are correlative and do not establish a direct regulatory mechanism. Furthermore, because fertility outcomes were not evaluated, the potential implications of these molecular changes for male fertility remain undetermined. Future investigations incorporating in vitro embryo production and embryo transfer, together with advanced molecular and epigenetic analyses, are warranted to validate these findings and further elucidate the underlying biological mechanisms.

## Figures and Tables

**Table 1:** The mean (±SEM) of spermatological parameters of post-thaw ram semen in Nicotinic acid (NA) and different doses of Nicotinic acid/Folic acid combination (NAFA) groups.

Groups			Spermatological Parameters			
	*n*	Total Motility (%)	Plasma Membrane Integrity (%)	Acrosomal Membrane Damage (%)	Low Mitochondrial Membrane Potential (%)	DNA Fragmentation (%)
Control	5	43.44 ± 4.07	66.80 ± 0.97	48.00 ± 2.59 ^a^	13.80 ± 3.77 ^a^	10.20 ± 1.39
NA10	5	49.58 ± 6.30	70.60 ± 2.40	41.20 ± 2.87 ^ab^	5.30 ± 0.97 ^b^	7.74 ± 1.65
NA10FA50	5	53.34 ± 9.51	73.00 ± 4.69	36.50 ± 4.17 ^b^	4.25 ± 1.03 ^b^	8.25 ± 2.02
NA20FA50	5	44.96 ± 2.20	65.75 ± 2.84	43.00 ± 4.14 ^ab^	4.50 ± 1.19 ^b^	9.00 ± 2.27

^a,b^ Different letters within the same column show significant differences among the groups (*p* < 0.05; *n*: replicates). Control, 10 mM NA with no FA (NA10), 10 mM NA + 50 nM FA (NA10FA50) and 20 mM NA + 50 nM FA (NA20FA50).

**Table 2:** The mean (±SEM) of Computer-Assisted Sperm Analysis (CASA) kinematics of post-thaw ram semen in Nicotinic acid (NA) and different doses of Nicotinic acid/Folic acid combination (NAFA) groups.

Groups	CASA Kinematic Parameters											
	*n*	ALH (μm)	BCF (Hz)	DAP (μm/s)	DCL (μm/s)	DSL (μm/s)	LIN (%)	STR (%)	VAP (μm/s)	VCL (μm/s)	VSL (μm/s)	WOB (%)
Control	5	6.01 ± 0.61 ^a^	34.93 ± 1.19 ^a^	36.79 ± 4.88 ^a^	63.80 ± 9.71 ^a^	30.26 ± 3.74 ^a^	60.35 ± 4.47 ^a^	85.87 ± 1.63 ^a^	80.30 ± 10.00 ^a^	139.38 ± 20.34 ^a^	66.24 ± 7.58 ^a^	68.30 ± 4.13 ^a^
NA10	5	3.04 ± 0.78 ^ab^	30.94 ± 0.73 ^b^	25.10 ± 5.05 ^ab^	38.32 ± 9.88 ^ab^	21.17 ± 4.27 ^ab^	76.06 ± 3.51 ^b^	91.51 ± 1.08 ^b^	53.61 ± 10.62 ^b^	81.95 ± 20.76 ^ab^	45.17 ± 9.05 ^ab^	80.76 ± 3.08 ^b^
NA10FA50	5	3.97 ± 0.73 ^ab^	38.04 ± 6.49 ^ab^	30.20 ± 2.99 ^ab^	44.14 ± 6.73 ^a^	25.99 ± 2.66 ^a^	72.44 ± 4.89 ^ab^	90.08 ± 2.13 ^ab^	66.93 ± 7.55 ^ab^	99.01 ± 17.32 ^a^	57.51 ± 6.45 ^a^	78.39 ± 4.03 ^ab^
NA20FA50	5	2.96 ± 0.21 ^b^	30.15 ± 0.58 ^b^	22.00 ± 1.37 ^b^	31.99 ± 1.55 ^b^	18.04 ± 1.43 ^b^	75.22 ± 2.75 ^ab^	90.21 ± 1.28 ^ab^	48.84 ± 3.06 ^b^	71.37 ± 3.44 ^b^	40.22 ± 3.13 ^b^	81.18 ± 1.99 ^ab^

^a,b^ Different letters within the same column show significant differences among the groups (*p* < 0.05). Control, 10 mM NA with no FA (NA10), 10 mM NA + 50 nM FA (NA10FA50) and 20 mM NA + 50 nM FA (NA20FA50). ALH: amplitude of lateral head displacement; BCF: beat cross frequency; DAP: average path distance; DCL: curvilinear distance; DSL: straight-line distance; LIN: linearity; STR: straightness; VAP: average path velocity; VCL: curvilinear velocity; VSL: straight-line velocity; WOB: wobble.

**Table 3:** The mean (±SEM) antioxidant properties (malondialdehyde concentration (MDA), total antioxidant capacity (TAC)) of post-thaw ram semen in Nicotinic acid (NA) and different doses of Nicotinic acid/Folic acid combination (NAFA) groups.

Groups		Antioxidant Properties	
	*n*	MDA (nmol/mL)	TAC (U/mL)
Control	5	2.97 ± 0.72	16.08 ± 3.20
NA10	5	3.37 ± 0.36	16.60 ± 4.16
NA10FA50	5	3.07 ± 0.57	15.66 ± 3.83
NA20FA50	5	3.33 ± 0.72	16.03 ± 1.42

No significant differences among the groups (*p* > 0.05). Control, 10 mM NA with no FA (NA10), 10 mM NA + 50 nM FA (NA10FA50) and 20 mM NA + 50 nM FA (NA20FA50). MDA: malondialdehyde; TAC: total antioxidant capacity.

**Table 4:** Primer sequences (5′-3′) and amplification characteristics used for quantitative real-time PCR analysis.

Gene	Forward Primer	Reverse Primer	Amplicon Size (bp)	Tm (°C)	GC (%)
*OGG1*	CAGACTGAGGAGCATCTCTACT	TACTGTTGCACGGCCTTTAG	95	58.45/58.20	50.0/50.0
*ROMO1*	TTCCTGTCTCAGGATTGGAATG	GGCCATGAATGTGCCAAAG	97	57.77/57.55	45.45/52.63
*ACTB* *	TCTTCCAGCCTTCCTTCCT	TAGAGGTCCTTGCGGATGT	100	57.50/57.70	52.63/52.63
*GAPDH* *	TGAGATCAAGAAGGTGGTGAAG	GCATCGAAGGTAGAAGAGTGAG	123	57.72/58.30	45.45/50.00

* Housekeeping gene.

**Table 5:** Primer sequences (5′-3′) utilized in the study for both methylated and unmethylated states.

Primer Type	*OGG1*	*ROMO1*
Left methylated primer	CGGGTAGATAGGTTTTGAGGTATC	TTAGGGTTTTGAGGGTTTGC
Right methylated primer	GATTAACGCGAACTAAACTCG	CGAAACTTTCTCCTAAACTAAACGA
Left unmethylated primer	GTGGGTAGATAGGTTTTGAGGTATT	ATTTTAGGGTTTTGAGGGTTTGT
Right unmethylated primer	ACAATTAACACAAACTAAACTCACT	AAAACTTTCTCCTAAACTAAACAAC

## Data Availability

The data supporting the findings of this study are available from the corresponding author upon reasonable request.

## Supplementary Materials

The following supporting information can be downloaded at: https://www.mdpi.com/article/10.3390/ijms27188422/s1.

## Abbreviations

The following abbreviations are used in this manuscript:

ACTB	Actin Beta
ALH	Amplitude of Lateral Head Displacement
ANOVA	Analysis of Variance
BCF	Beat Cross Frequency
BER	Base Excision Repair
BLAST	Basic Local Alignment Search Tool
CASA	Computer-Assisted Sperm Analysis
cDNA	Complementary DNA
DAP	Distance Average Path
DCL	Distance Curved Line
DNA	Deoxyribonucleic Acid
DSL	Distance Straight Line
EDTA	Ethylenediaminetetraacetic Acid
ELISA	Enzyme-Linked Immunosorbent Assay
FA	Folic Acid
FITC	Fluorescein Isothiocyanate
FITC-PSA	Fluorescein Isothiocyanate-Conjugated Pisum sativum Agglutinin
GAPDH	Glyceraldehyde-3-Phosphate Dehydrogenase
HOST	Hypoosmotic Swelling Test
HRP	Horseradish Peroxidase
JC-1	5,5′,6,6′-Tetrachloro-1,1′,3,3′-Tetraethylbenzimidazolylcarbocyanine Iodide
LIN	Linearity
MDA	Malondial-dehyde
MMP	Mitochondrial Membrane Potential
mRNA	Messenger RNA
NA	Nicotinic Acid
NAD	Nicotinamide Adenine Dinucleotide
NADP	Nicotinamide Adenine Dinucleotide Phosphate
NA-FA	Nicotinic Acid/Folic Acid Combination
NCBI	National Center for Biotechnology Information
OD	Optical Density
OGG1	8-Oxoguanine DNA Glycosylase 1
OS	Oxidative Stress
PCR	Polymerase Chain Reaction
PSA	Pisum sativum Agglutinin
RNA	Ribonucleic Acid
ROMO1	Reactive Oxygen Species Modulator 1
ROS	Reactive Oxygen Species
SEM	Standard Error of the Mean
SPSS	Statistical Package for the Social Sciences
STR	Straightness
TAC	Total Antioxidant Capacity
TCA	Tricarboxylic Acid
TM	Total Motility
TUNEL	Terminal Deoxynucleotidyl Transferase-Mediated dUTP Nick-End Labeling
VAP	Average Path Velocity
VCL	Curvilinear Velocity
VSL	Straight-Line Velocity
WOB	Wobble
mM	milimolar
nM	nanomolar
