# Supplementary material for: Nicotinic and Folic Acid Supplementation Improves Cryopreserved Ram Sperm Quality, Potentially Through Modulation of OGG1 and ROMO1 Expression and DNA Methylation
Source: Int J Mol Sci. 2026 Sep 21;27(18):8422. doi: 10.3390/ijms27188422 (PMC13607251; doi:10.3390/ijms27188422)
Supplement: Supplementary file 1 [file ijms-27-08422-s001.zip › Figure S1.pdf]

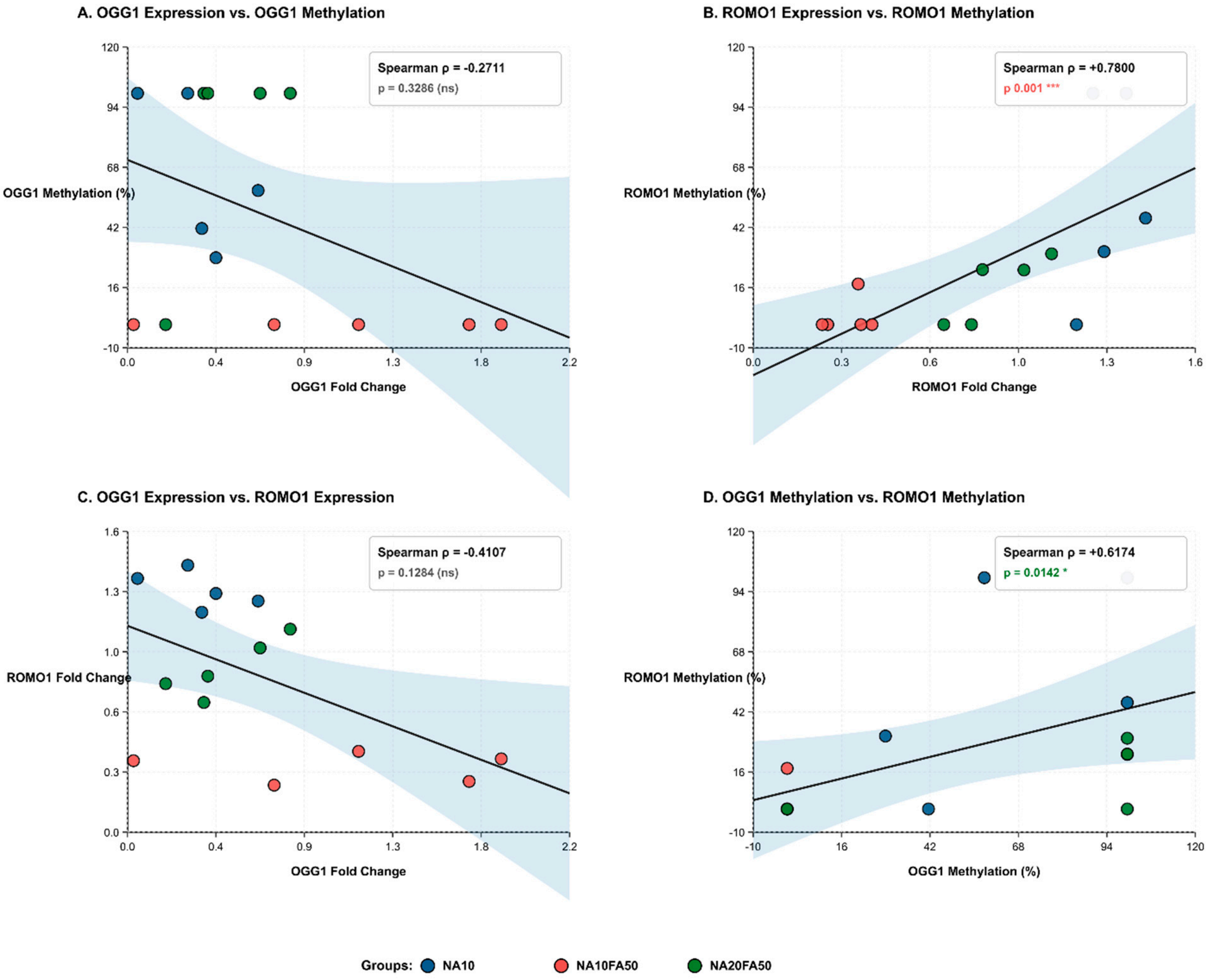

Figure S1. Correlation analysis of *OGG1* and *ROMO1* gene expression and DNA methylation levels. Spearman rank correlation analyses were performed to evaluate the relationships between (A) *OGG1* expression (fold change) and OGG1 methylation (%), (B) *ROMO1* expression (fold change) and *ROMO1* methylation (%), (C) *OGG1* and *ROMO1* expression levels (fold change), and (D) *OGG1* and *ROMO1* methylation levels (%). Each point represents an individual observation, with colors indicating the experimental groups: NA10 (blue), NA10FA50 (red), and NA20FA50 (green). Solid black lines represent fitted linear regression lines, and shaded areas indicate the corresponding 95% confidence intervals. Spearman's correlation coefficients (ρ) and associated p-values are shown within each panel. Statistical significance was defined as $p < 0.05$; $p < 0.01$ is indicated by **. ns, not significant. NA, nicotinic acid; FA, folic acid.
