# Supplementary material for: Nicotinic and Folic Acid Supplementation Improves Cryopreserved Ram Sperm Quality, Potentially Through Modulation of OGG1 and ROMO1 Expression and DNA Methylation
Source: Int J Mol Sci. 2026 Sep 21;27(18):8422. doi: 10.3390/ijms27188422 (PMC13607251; doi:10.3390/ijms27188422)
Supplement: Supplementary file 1 [file ijms-27-08422-s001.zip › Table S1.pdf]

**Table S1.** Kruskal–Wallis test statistics (H) and corresponding effect size estimates (epsilon squared, $\varepsilon^2$) for *OGG1* and *ROMO1* gene expression (fold change) and promoter methylation analyses.

| **Parameter** | **H** | **$\varepsilon^2$** |
|---|---|---|
| *OGG1* fold change | 3.440 | 0.120 |
| *ROMO1* fold change | 12.500 | 0.875 |
| *OGG1* methylation | 8.794 | 0.566 |
| *ROMO1* methylation | 6.502 | 0.375 |
